# Integrating Network Pharmacology Approaches to Decipher the Multi-Target Pharmacological Mechanism of Microbial Biosurfactants as Novel Green Antimicrobials against Listeriosis

**DOI:** 10.3390/antibiotics12010005

**Published:** 2022-12-20

**Authors:** Mohd Adnan, Arif Jamal Siddiqui, Emira Noumi, Sami Hannachi, Syed Amir Ashraf, Amir Mahgoub Awadelkareem, Mejdi Snoussi, Riadh Badraoui, Fevzi Bardakci, Manojkumar Sachidanandan, Mirav Patel, Mitesh Patel

**Affiliations:** 1 Department of Biology, College of Science, University of Hail, Hail P.O. Box 2440, Saudi Arabia; 2 Department of Clinical Nutrition, College of Applied Medial Sciences, University of Hail, Hail P.O. Box 2440, Saudi Arabia; 3 Section of Histology-Cytology, Medicine Faculty of Tunis, University of Tunis El Manar, La Rabta 1007, Tunis, Tunisia; 4 Department of Oral Radiology, College of Dentistry, University of Hail, Hail P.O. Box 2440, Saudi Arabia; 5 Department of Biotechnology, Parul Institute of Applied Sciences and Centre of Research for Development, Parul University, Vadodara 391760, India

**Keywords:** *Listeria monocytogenes*, listeriosis, network pharmacology, biosurfactants, antimicrobial

## Abstract

*Listeria monocytogenes* (*L. monocytogenes*) is a serious food-borne pathogen that can cause listeriosis, an illness caused by eating food contaminated with this pathogen. Currently, the treatment or prevention of listeriosis is a global challenge due to the resistance of bacteria against multiple commonly used antibiotics, thus necessitating the development of novel green antimicrobials. Scientists are increasingly interested in microbial surfactants, commonly known as “biosurfactants”, due to their antimicrobial properties and eco-friendly nature, which make them an ideal candidate to combat a variety of bacterial infections. Therefore, the present study was designed to use a network pharmacology approach to uncover the active biosurfactants and their potential targets, as well as the signaling pathway(s) involved in listeriosis treatment. In the framework of this study, 15 biosurfactants were screened out for subsequent studies. Among 546 putative targets of biosurfactants and 244 targets of disease, 37 targets were identified as potential targets for treatment of *L. monocytogenes* infection, and these 37 targets were significantly enriched in a Gene Ontology (GO) analysis, which aims to identify those biological processes, cellular locations, and molecular functions that are impacted in the condition studied. The obtained results revealed several important biological processes, such as positive regulation of MAP kinase activity, protein kinase B signaling, ERK1 and ERK2 cascade, ERBB signaling pathway, positive regulation of protein serine/threonine kinase activity, and regulation of caveolin-mediated endocytosis. Several important KEGG pathways, such as the ERBBB signaling pathway, TH17 cell differentiation, HIF-1 signaling pathway, *Yersinia* infection, Shigellosis, and C-type lectin receptor signaling pathways, were identified. The protein–protein interaction analysis yielded 10 core targets (IL2, MAPK1, EGFR, PTPRC, TNF, ITGB1, IL1B, ERBB2, SRC, and mTOR). Molecular docking was used in the latter part of the study to verify the effectiveness of the active biosurfactants against the potential targets. Lastly, we found that a few highly active biosurfactants, namely lichenysin, iturin, surfactin, rhamnolipid, subtilisin, and polymyxin, had high binding affinities towards IL2, MAPK1, EGFR, PTPRC, TNF, ITGB1, IL1B, ERBB2, SRC, and mTOR, which may act as potential therapeutic targets for listeriosis. Overall, based on the integrated network pharmacology and docking analysis, we found that biosurfactants possess promising anti-listeriosis properties and explored the pharmacological mechanisms behind their effect, laying the groundwork for further research and development.

## 1. Introduction

More than 200 diseases can be caused in humans by food-borne contaminations, which are caused by a variety of factors that are involved with the cause and development of disease related to food consumption [1]. In this regard, we can point to the increasing population of the world, which has led to the subsequent rise in the demand for food, as well as microbial genomic diversification and selection pressures, resulting in the emergence of new pathogens as a result of the growing popularity of eating outside the home [2]. An infection caused by the bacterium *Listeria monocytogenes* (*L. monocytogenes*) is called “listeriosis” and is usually a result of eating food that has been contaminated with this food pathogen. In a wide range of food products, such as dairy products, raw vegetables, and raw meat, as well as ready-to-eat products, this bacterium has been found to be present [3]. The *L. monocytogenes* are a Gram-positive, rod-shaped, non-spore forming, non-capsule forming bacteria, which are motile at 10 to 25 °C [4]. They can infect a wide range of human and animals cell types. Few populations of humans are reported to carry the bacterium without showing symptoms in the intestinal tract [5]. Following the ingestion of bacterium by the host, *L. monocytogenes* first encounters epithelial cells of the gut lining and then enters the host’s monocytes, macrophages, or polymorphonuclear leukocytes. The bacterium becomes blood-borne and multiplies both intracellularly and extracellularly. In pregnant women, it can migrate through the placenta to reach the fetus intracellularly [6]. When *L. monocytogenes* is infected in mice, the bacteria first appear in macrophages before spreading to liver hepatocytes [7]. Several outbreaks have been associated with the consumption of ready-to-eat food, because *L. monocytogenes* is capable of growing at refrigerated temperatures [8]. 

There are several high-risk populations that are susceptible to listeriosis, including the elderly, pregnant women, newborns, and immunocompromised patients due to kidney transplant, cancer, HIV/AIDS, and steroid therapy [8]. Around the world, there are approximately 1600 cases of listeriosis each year, and approximately 260 people die from it [9]. Despite the fact that there are a small number of cases of listeriosis in the world, the high rate of death associated with this infection makes it an important public health concern. Due to this, there is a need to implement effective medical management for listeriosis. Therefore, alternative measures are needed to control *L. monocytogenes* in the food industry.

Over the past few years, natural products and their derivatives have been gaining more and more attention as insights into research and possible drug sources for targeted therapy, owing to their variety of structural features, multi-target action, and low toxicity [10]. There have been a great number of dramatic advances in high-throughput screening techniques over the past few decades that have greatly contributed to the discovery of novel drugs based on natural products [11]. Hence, a new discovery of a potential bioactive compound that can affect the pathophysiology of diseases and disorders will be considered a thunderbolt of this new era of pharmaceuticals. 

Biological surfactants (biosurfactants) are surface active compounds which are synthesized by the microbes (bacteria and fungi) on their cell surface or excreted that can reduce surface and interfacial tension [12]. There is no doubt that biosurfactants are becoming more and more popular among scientists because of their eco-friendliness properties, scalability, durability under harsh environmental conditions, specificity, and versatility, which make them appealing for their application in various fields [13]. There are numerous applications for these compounds as antimicrobials, anti-adhesives, and anticancer agents, in addition to being extensively used for the purposes of recovery of oil, bioremediation, and emulsification in industry [14]. 

**Figure 1:**
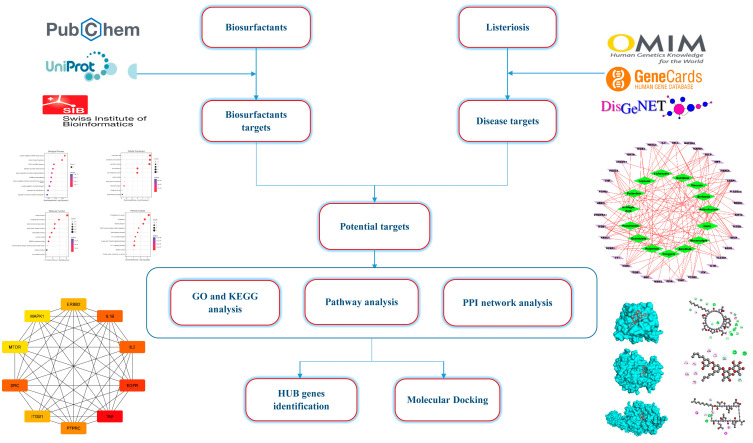
Framework based on an integration strategy of network pharmacology.

In previous studies, biosurfactants have been demonstrated to have antimicrobial, antibiofilm, and anti-listeriosis properties [13,14,15], suggesting that they could potentially be useful for preventing and treating listeriosis. In spite of this, very few studies have been published that have examined the use of biosurfactants in the prevention and treatment of listeriosis, and no research has examined the mechanisms behind their action [15]. Insights into the mechanisms of action of biosurfactants against listeriosis will be possible if studies focusing on molecular targets and their related signal pathways are conducted. To accomplish this purpose, we utilized network pharmacology [16,17] and a molecular docking methodology [18] approach in the present study to construct a multidimensional network of “component–target–pathway–disease” that is able to explain the biological mechanisms underlying biosurfactants for the prevention and treatment of listeriosis. It is intended that the results of the present study will provide a scientific foundation for clinical trial research and the development of biosurfactant products in the future. Figure 1 illustrates the flowchart of this study.

## 2. Results

### 2.1. Identification of Active Components of Biosurfactants

**Figure 2:**
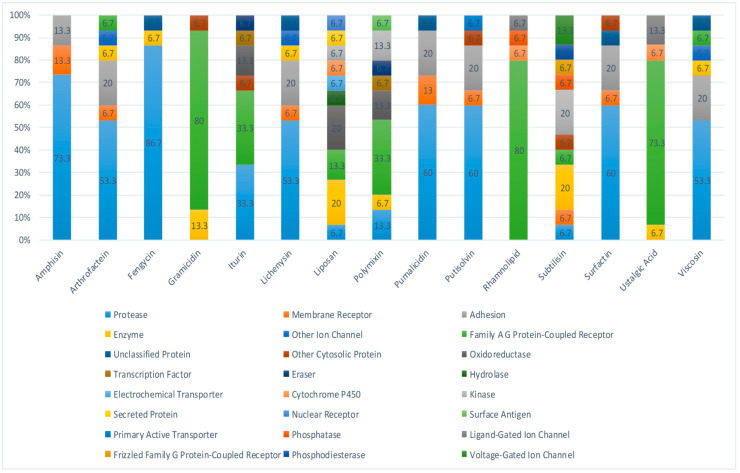
Biosurfactants and their potential proteins interaction, as retrieved from the SwissTargetPrediction server.

**Figure 3:**
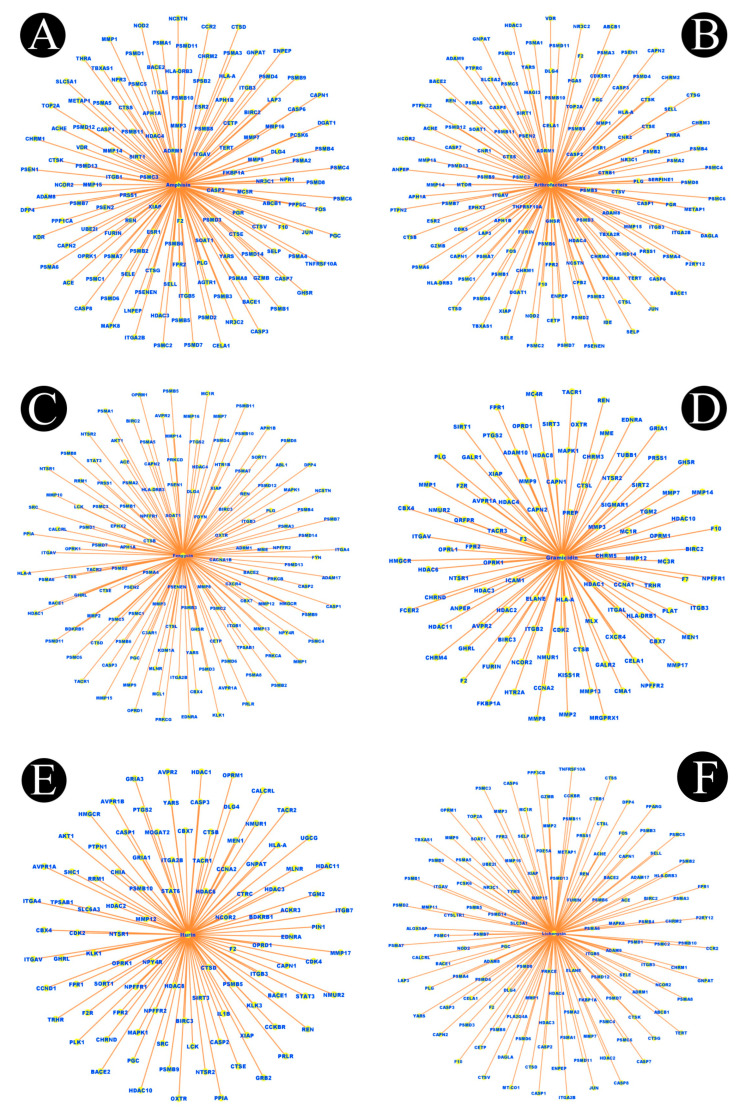
Biosurfactants and their potential target networks: (**A**) amphisin, (**B**) arthrofactin, (**C**) fengycin, (**D**) gramicidin, (**E**) iturin, and (**F**) lichenysin. Edges (orange color) represent respective protein targets.

**Figure 4:**
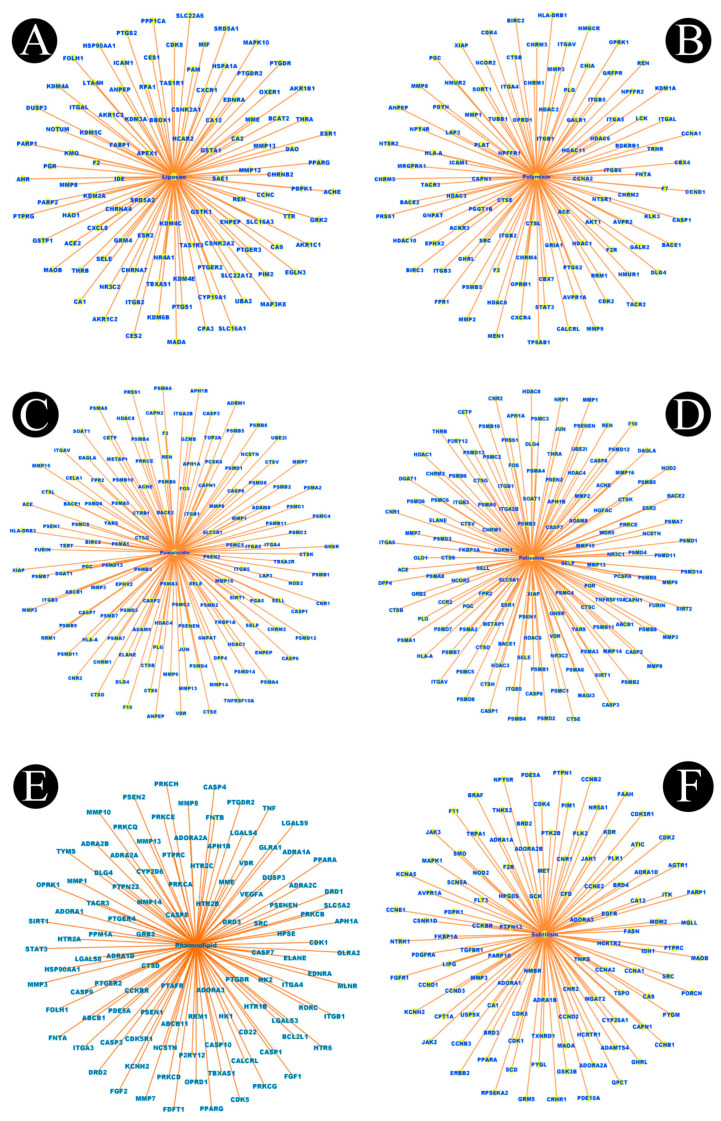
Biosurfactants and their potential target networks. (**A**) liposan, (**B**) polymyxin, (**C**) pumalicidin, (**D**) putisolvin, (**E**) rhamnolipid, and (**F**) subtilisin. Edges (orange color) represent respective protein targets.

**Figure 5:**
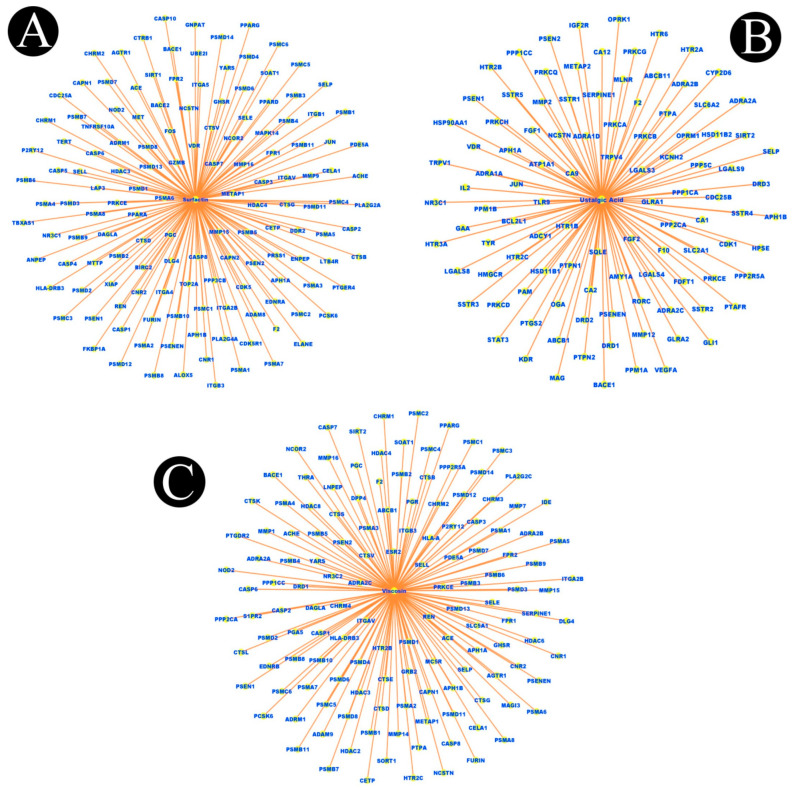
Biosurfactants and their potential target networks. (**A**) surfactin, (**B**) ustilagic acid, and (**C**) viscosin. Edges (orange color) represent respective protein targets.

**Figure 6:**
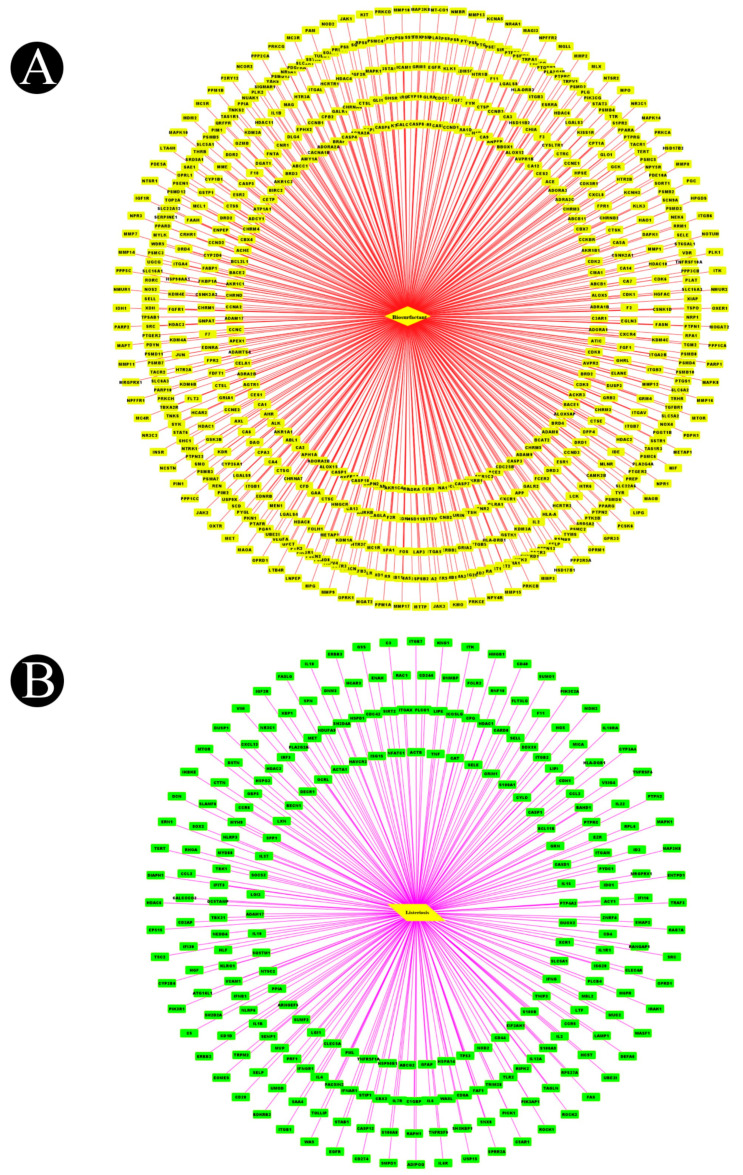
(**A**) Biosurfactant–gene network after removing duplication of genes (diamond indicates biosurfactants and rectangle indicates target proteins). (**B**). Disease–gene network after removing duplication of genes (parallelogram indicates disease, and rectangle indicates target proteins).

In total, 15 biosurfactants were selected, and their detailed information was retrieved from the PubChem database in order to be analyzed, using the SwissTargetPrediction database (Table 1). We predicted the potential protein targets of each biosurfactant by using SwissTargetPrediction (Figure 2A–F, Figure 3A–F, Figure 4A–F, and Figure 5A–C). Following the removal of duplicate targets from the target prediction, screening of 546 potential targets was conducted for further evaluation. A visual compound–target network was subsequently constructed by using Cytoscape 3.9.1 in order to construct a visual network with 546 nodes and 545 edges (Figure 6A). The nodes represent ingredients and their corresponding targets. The higher the degree corresponding to the node, the greater the pharmacological effects of this ingredient or target. The calculated average shortest path length, betweenness centrality, closeness centrality, and degree of nodes in the network are shown in Table 2. 

### 2.2. Listeriosis and Intersection Target

**Figure 7:**
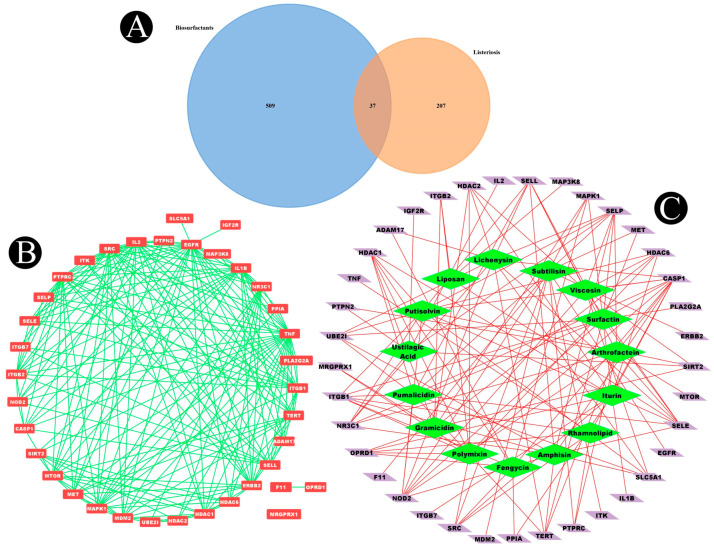
(**A**) Venn diagram showing common genes between listeriosis and biosurfactants. (**B**) Interconnected common genes network constructed by using Cytoscape. (**C**) Biosurfactants–common-genes target network (diamond indicates biosurfactants and parallelogram indicates common target proteins).

The human genome database was used to collect the targets that are related to listeriosis. A total of 197, 276, and 211 targets were identified in the OMIM, DisGeNET, and GeneCard databases, respectively. As a result of removing duplicate entries from these three kinds of databases, a total of 244 listeriosis targets were obtained (Figure 6B). By intersecting these targets with component targets, a total of 37 intersection targets were obtained, as shown in Figure 7A. Figure 7B,C show a diagram of component intersection targets that has 52 nodes and 133 edges that were created with the help of Cytoscape.

### 2.3. Construction of Protein–Protein Interaction Network (PPI) and Key Targets

**Figure 8:**
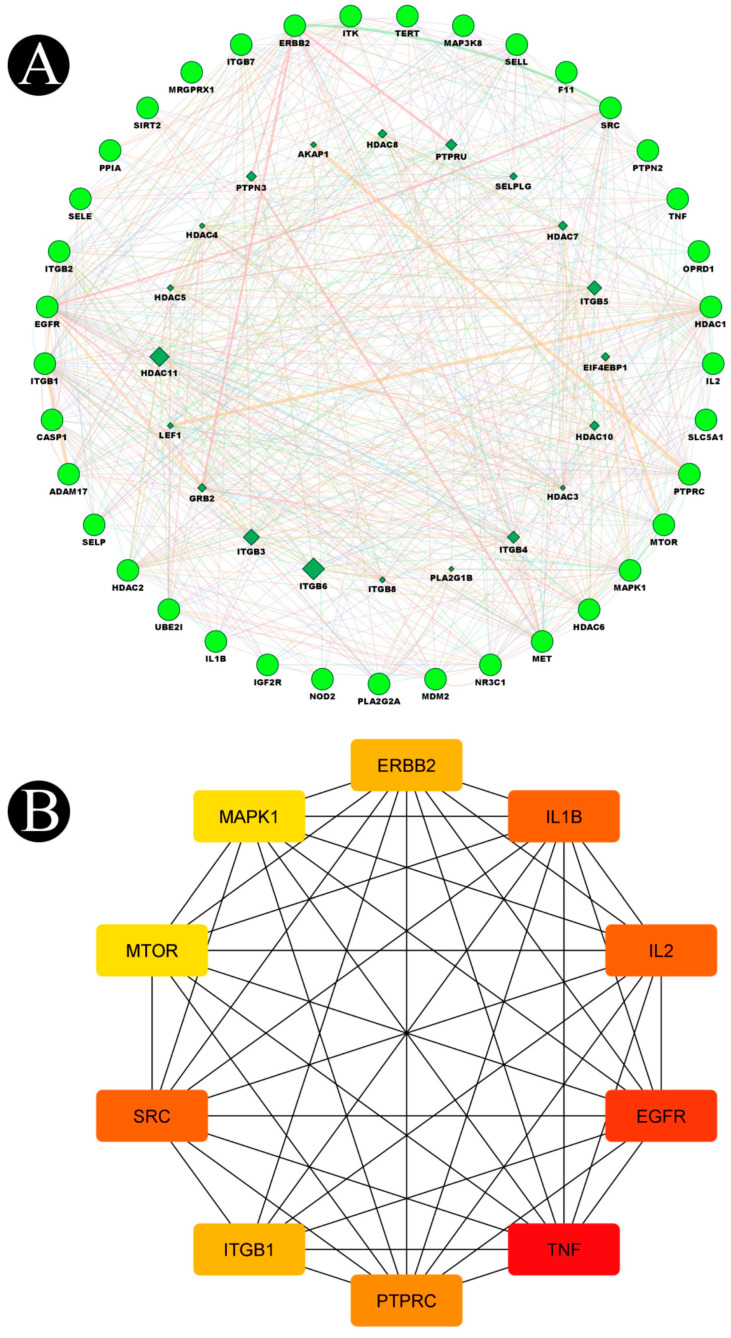
(**A**) Network of potential targets of biosurfactants against listeriosis analyzed by GeneMANIA. Genes on the outer ring were submitted as query terms in searches. Nodes on the inner ring indicate genes associated with query genes. Functional association of targets was analyzed, and different colors of connecting lines represent different correlations. (**B**) Key subnetwork of the top 10 nodes analyzed by CytoHubba.

Utilizing the GeneMANIA tool, we imported 37 target genes in order to obtain a PPI network that demonstrates the relationships between these 37 target genes and other genes in the network. In the results, the percentage represents the weight that is given to interaction relationships in the network. According to our results, 29.46% of the interactions between the targets in the network resulted in co-expressions, and 35.77% of them resulted in physical interactions. Furthermore, there was a relationship between co-localization and shared protein domains (Figure 8A). In Table 3, we provide the calculated average length of shortest paths to the three central nodes, betweenness centrality, closeness centrality, and degree of each node in the network. There were ten targets in the network which are organized in the order of high to low, according to the topology properties of the network, corresponding to EGFR, SRC, IL1B, IL2, PTPRC, ERBB2, ITGB1, MAPK1, MTOR, and TNF (Figure 8B). Biosurfactants may be able to prevent and treat listeriosis by targeting these ten targets, as they may be the key targets for biosurfactants.

### 2.4. Functional GO and KEGG Pathways

**Figure 9:**
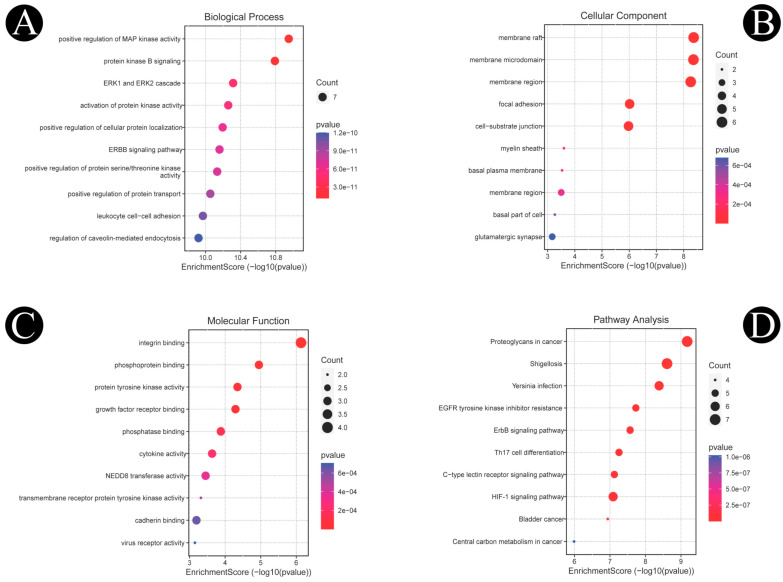
GO enrichment and KEGG pathway analyses of 37 target proteins (*p*-value ≤ 0.05). (**A**) The top 10 biological processes. (**B**) The top 10 cellular components. (**C**) The top 10 molecular functions. (**D**) The top 10 KEGG pathways. The color scales indicate the different thresholds for the *p*-values, and the sizes of the dots represent the number of genes corresponding to each term.

By using the Shiny GO 0.76.2 database analytical tool, the 37 intersected genes were enriched by GO and KEGG analysis. As a result of incorporating biological process (BP), molecular function (MF) and cellular component (CC) (Figure 9A–C), along with a *p*-value < 0.05, as screening conditions, a total of 1255 items were obtained pertaining to biological process, 149 items were obtained pertaining to molecular function, and 94 items were obtained pertaining to cellular composition. The hypothesis was put forth that biosurfactants could be involved in inhibiting listeriosis through the positive regulation of MAP kinase activity, protein kinase B signaling, ERK1 and ERK2 cascade, and the ERBB signaling pathway; positive regulation of protein serine/threonine kinase activity and leukocyte cell–cell adhesion; positive regulation of establishment of protein localization; and regulation of caveolin-mediated endocytosis via molecular functions such as integrin binding, phosphoprotein binding, protein tyrosine kinase activity, growth factor receptor binding, phosphatase binding, cytokine activity, NEDD8 transferase activity, and cadherin binding in cellular compartments such as membrane raft, membrane microdomain, focal adhesion, cell–substrate junction, myelin sheath, basal plasma membrane, and basal part of the cell. A total of 190 enrichment results were obtained from the KEGG pathway enrichment analysis. Among them Shigellosis, *Yersinia* infection, the ERBB signaling pathway, Th17 cell differentiation, the HIF-1 signaling pathway, the C-type lectin receptor signaling pathway, and bladder cancer pathways are closely associated with listeriosis and are in accordance with the enrichment results of GO. There was a significant abundance of KEGG pathways and gene pathways with *p*-values ≤ 0.05. Based on the Shiny GO platform, the first ten components were analyzed (Figure 9D). Based on the statistical analysis, ten proteins exhibited a high frequency of participation in the first 10 pathways, indicating that they played a major role in the enrichment pathway. The ten core proteins are EGFR, SRC, IL1B, IL2, PTPRC, ERBB2, ITGB1, MAPK1, MTOR, and TNF.

### 2.5. Molecular Docking

**Figure 10:**
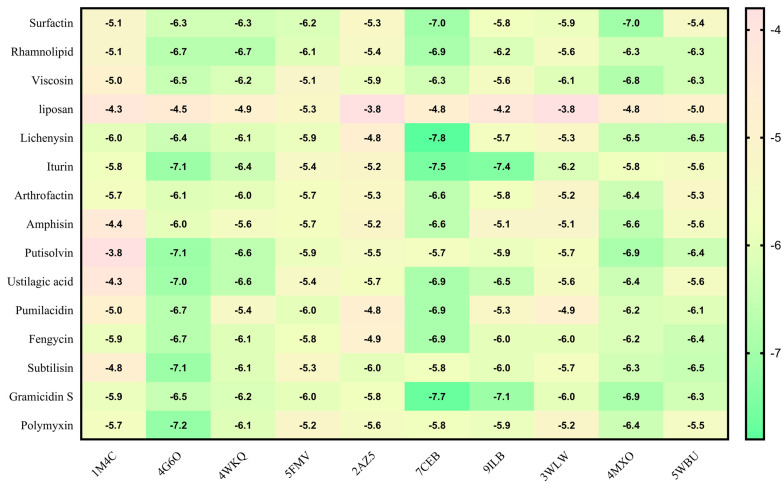
Binding affinities of top-rated pose of ligand–receptor complex.

**Figure 11:**
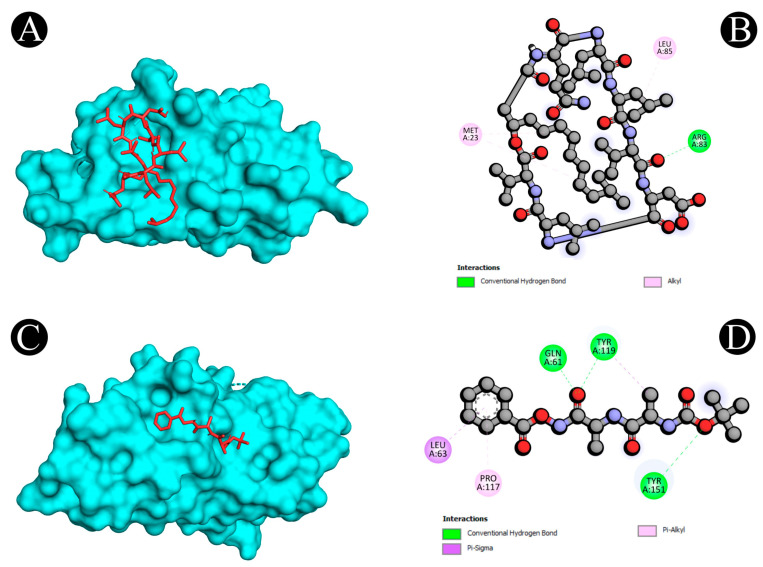
(**A**,**B**) Visualization of docking analysis of IL2 and lichenysin. (**C**,**D**) Visualization of docking analysis of TNF and subtilisin.

**Figure 12:**
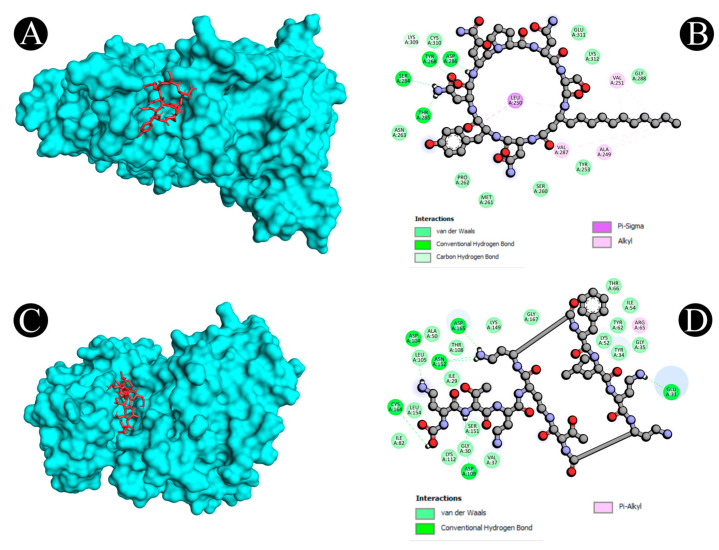
(**A**,**B**). Visualization of docking analysis of ERBB2 and iturin. (**C**,**D**) Visualization of docking analysis of MAPK1 and polymyxin.

**Figure 13:**
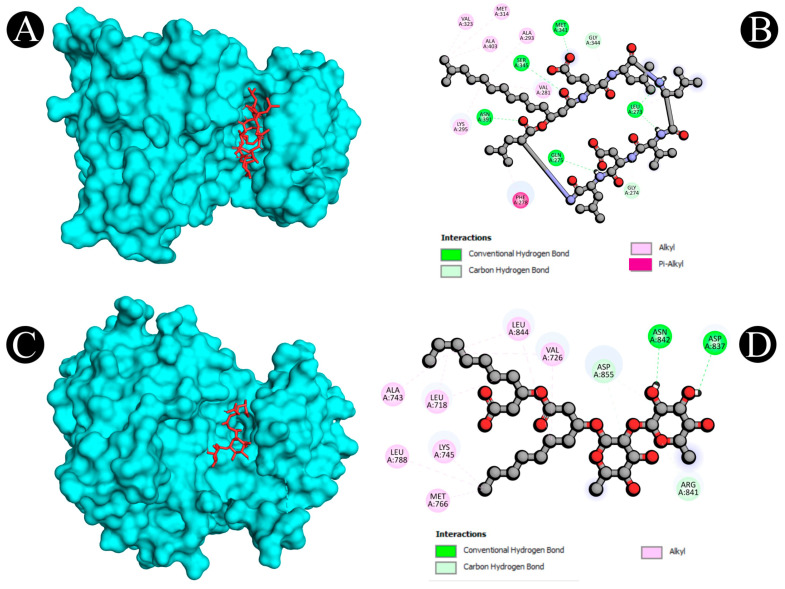
(**A**,**B**) Visualization of docking analysis of SRC and surfactin. (**C**,**D**) Visualization of docking analysis of EGFR and rhamnolipid.

**Figure 14:**
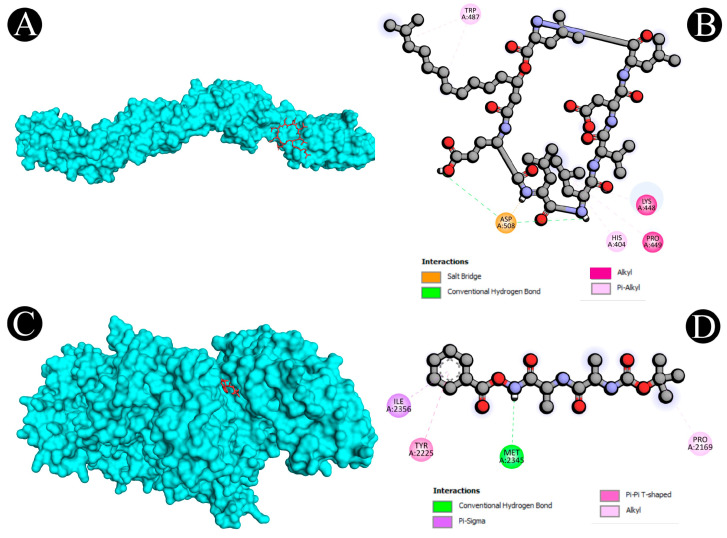
(**A**,**B**) Visualization of docking analysis of PTPRC and surfactin. (**C**,**D**) Visualization of docking analysis of MTOR and subtilisin.

**Figure 15:**
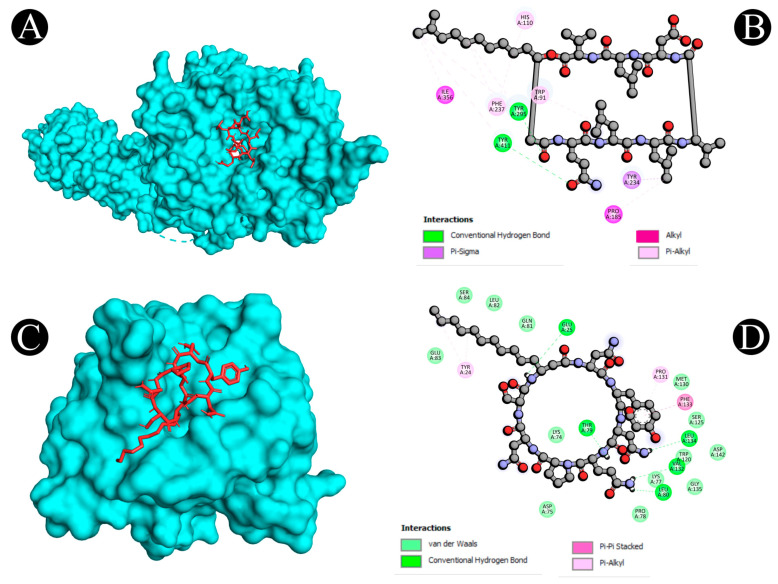
(**A**,**B**) Visualization of docking analysis of ITGB1 and lichenysin. (**C**,**D**) Visualization of docking analysis of IL1B and iturin.

Virtual screening using molecular docking is a computational method for identifying potential leads against predefined targets. By employing this method, compounds with appreciable binding affinities and specific interactions with target proteins were identified. A docking analysis of all the biosurfactants revealed the presence of several compounds with a significant affinity for the respective target proteins (Figure 10). The highest binding affinity was found between IL2–lichenysin (−6.0 kJ/mol), MAPK1–polymyxin (−7.2 kJ/mol), EGFR–rhamnolipid (−6.7 kJ/mol), PTPRC–surfactin (−6.2 kJ/mol), TNF–subtilisin (−6.0 kJ/mol), ITGB1–lichenysin (−7.8 kJ/mol), IL1B–iturin (−7.4 kJ/mol), ERBB2–iturin (−6.2 kJ/mol), SRC–surfactin (−7.0 kJ/mol), and mTOR–subtilisin (−6.5 kJ/mol). These results suggest that the few selected biosurfactants have a significant level of binding efficiency with respective proteins, which may contribute to the development of a potential binding partner for selective proteins that could be used for drug development. The best biosurfactants observed occupying the active site in different ways can be seen in Figure 11, Figure 12, Figure 13, Figure 14 and Figure 15 and Table 4.

## 3. Discussion

Over the past 80 years, *L. monocytogenes* has been identified as a human pathogen that has the potential to cause disease. There has been a demographic shift in the last few decades, and there has been an explosion of immunosuppressive medications used for treating malignancies and managing organ transplants. This has led to an increasing number of immunocompromised individuals who are at an increased risk for listeriosis [34]. There is also the factor of changing consumer lifestyles which has resulted in less time available for food preparation, as well as an increase in the use of ready-to-eat food and take-away food. Food production and technology have drastically changed in recent years, resulting in foods with longer shelf-lives that are considered to be “Listeria-risk foods”; the bacteria multiply for a longer period of time, so the food does not undergo a listericidal process before consumption [35]. A high case-fatality rate of between 20 and 30% has been reported for listeriosis, compared to other common food-borne pathogens. During the past three decades, an epidemiological investigation has suggested that epidemic and sporadic listeriosis are primarily linked with the ingestion of foods or food products that are contaminated. While listeriosis is a rare food-borne illness in comparison to other food-borne illnesses, it is a very serious one. There is a high mortality rate associated with the disease even with adequate antibiotic treatment. Approximately, 90% of patients who have listeriosis are hospitalized, and many of them are in intensive care units. As a result, listeriosis is a serious problem around the world. Presently, listeriosis remains a significant challenge, and current treatment options are not adequate to combat the disease [36].

A number of biosurfactants have been tested for their antimicrobial activity, which has shown to be effective against different types of bacterial pathogens, such as *Clostridium perfringens*, *Bacillus subtilis*, *Staphylococcus aureus*, etc. (Gram-positive bacteria); *Escherichia coli*, *Enterobacter aerogenes*, *Salmonella Typhimurium*, etc. (Gram-negative bacteria); and *Mucor* sp., *Phytophthora capsici*, *Fusarium graminearum*, *Botrytis cinerea*, and *Phytophthora infestans* (pathogenic fungi) [37,38]. There is no complete understanding of how these compounds exert their antimicrobial activity; however, one of their proposed sites of action is the cell membrane since they are amphipathic and thus can interact with phospholipids [39].

Moreover, to date only one study is published on the anti-listeriosis activity of biosurfactants. According to the report published by de Araujo et al. [40], there is evidence that *P. aeruginosa* PA1 produces rhamnolipids that have antibacterial activity against *L. monocytogenes* ATCC 19112 and ATCC 7644. In addition to screening microbial surfactants for the treatment of listeriosis, the present study identifies a new therapeutic concept for further investigation into the mechanism of biosurfactants. For complex diseases such as listeriosis, in terms of predictive analysis, network pharmacology offers unique advantages [41]. Analyzing the PPI network, 10 core targets, namely EGFR, SRC, IL1B, IL2, PTPRC, ERBB2, ITGB1, MAPK1, MTOR, and TNF, for biosurfactants against listeriosis were screened out in the present study. EGF (epidermal growth factor) receptors are tyrosine kinases that bind ligands from the EGF family and activate signaling cascades in order to convert extracellular signals into appropriate cellular responses. In order to induce endocytosis, *L. monocytogenes* interacts with this tyrosine kinase receptor and E-cadherin, which might be a common pathogen invasion mechanism for the entry of *L. monocytogenes* [42]. SRC (proto-oncogene tyrosine-protein kinase Src) is reported as closely related with the pathogenicity of *L. monocytogenes*. As a result of *L. monocytogenes* infection, the heavy chain of non-muscle myosin IIA (NMHC-IIA) is phosphorylated at a specific tyrosine residue [43]. The pro-inflammatory cytokine interleukin-1beta (IL-1β) has been known to have a protective function against a variety of bacterial, fungal, and viral infections [44]. Bacterial pathogens are capable of exploiting host cell signaling pathways in order to adhere to or internalize host cells. A frequent molecular alteration involves the phosphorylation of tyrosine kinases on host non-receptors and receptors [45]. 

Bacteria can induce phosphorylation through direct contact with host cells or via soluble factors [45]. In order to infect host cells, *L. monocytogenes* reported activating the ERBB2/ERBB3 heterodimer pathway [46]. Pathogens such as *Yersinia pseudotuberculosis*, *Staphylococcus aureus*, *Neisseria* species, and enteroaggregative *Escherichia coli* exploit the Integrin subunit beta 1 (ITGβ1) receptor for adhesion to or invasion of mammalian cells [47,48,49,50,51]. Additionally, another protein, mTOR (mammalian target of rapamycin), plays a crucial role in *Listeria* entry. Cell growth, autophagy, and actin cytoskeleton development are controlled by mTOR, a serine/threonine kinase that responds to growth factor stimulation and nutrient, energy, or oxygen availability. Further to this, MAPK family proteins are also reported to play a crucial role in the infection of *L. monocytogenes*, and therefore, the treatment of infection with MAPK inhibitors is reported to affect the inhibition of bacterial internalization towards the host cells during infection [52]. Additionally, tumor necrosis factor (TNF) is a cytokine that has also been reported to play an active role in the susceptibility to *L. monocytogenes* infection. The dysregulation of TNF production and function has been reported to be associated with *L. monocytogenes* pathogenesis since it plays a crucial role in inflammation [53]. The production of TNF is shown to contribute to the protection against *L. monocytogenes* infection in an experimental model, and it can also stimulate the production of IFN-γ [54]. In experiments on severe combined immunodeficiency mice infected with *L. monocytogenes*, it is also found to be involved in a T-cell-independent pathway that leads to macrophage activation [55]. Moreover, the contribution of TNF to the pathogenesis of *L. monocytogenes* has been shown when TNF- or TNF Receptor 1 (R1)-deficient mice succumbed to the *L. monocytogenes* infection relatively quickly instead of recovering after a few days like the control mice [56]. All of these protein targets which come out as a result of the present study can therefore be considered to be most important targets for treating *L. monocytogenes* infections in the future.

Based on the GO analysis, possible targets for biosurfactants against listeriosis are involved in multiple important GO processes. Human epidermal growth factor receptor tyrosine kinases have a size of around 180 kDa and are a family of candidate tyrosine kinase receptors [57]. This family of receptors (EGFR, also termed ERBB1/HER1, ERBB2 or neu/HER2, ERBB3 or HER3, and ERBB4 or HER4) is characterized by dimerization with other receptors that are either of the same nature (homodimerization) or of a different nature (heterodimerization) [58,59,60]. Interestingly, ERBB receptors play important roles in cancer development [61] and are also found in signaling between bacteria and their hosts. It has been shown that the binding of *Neisseria meningitidis* to endothelial cells leads to the clustering of ERBB2 receptors, followed by phosphorylation of receptor tyrosine and activation of downstream signaling molecules, leading to actin polymerization and bacterial internalization [62]. The envelope glycoprotein B of the human cytomegalovirus (HCMV) binds to EGFR and promotes its tyrosine phosphorylation upon heterodimerization with ErbB3, resulting in virus entry and viral protein synthesis [63]. Likewise, *L. monocytogenes* and other bacteria also trigger the activation of the ErbB2/ErbB3 heterodimer signaling pathway in order to invade host cells [46]. One more key protein that is utilized in classical endocytic mechanisms to allow various particles to be internalized is clathrin or caveolin [64,65,66]. In order to move between epithelial cells, *L. monocytogenes* hijacks the caveolin–endocytic machinery. The activation of these processes is mediated by a subset of caveolar proteins (caveolin-1, cavin-2, and EHD2). Moreover, it is well-known that pathogens manipulate the post-translational modifications (PTMs) of host proteins to interfere with the normal functioning of host cells in various ways. A key target among these modifications is ubiquitin (UBI), ubiquitin-like proteins (UBLs), and neural precursor cell expressed developmentally downregulated protein 8 (NEDD8), which regulate pathways necessary for the host cell. The PTM modifiers, for instance, regulate the pathways that are crucial to the spread of infection, such as the entry, replication, propagation, or detection of the pathogen by the host, which have all been linked to these PTM modifiers. Different enzymes are involved in this biological process, as well as molecular functions, such as protein kinase binding activity, and the reactions are occurring in a variety of locations, such as the membrane and cytoplasm [67]. There are several biological processes involved in this process that are mediated by different enzymes, along with molecular functions such as protein kinase activity, and all of these reactions occur in multiple locations, such as the membrane and the cytoplasm of the cell. Based on these findings, we suggested that biosurfactants might have an impact on these processes as a result obtained from the GO analysis in this study.

According to the KEGG pathways analysis, potential targets of biosurfactants against listeriosis are significantly enriched in several important pathways, such as the ERBB signaling pathway, C-type lectin receptor signaling, Th17 cell differentiation and HIF-1 signaling pathway, etc. As described above, the ERBB signaling pathway plays an important role in listeriosis to invade bacteria in host cells. A key role that dendritic cells play in tailoring immune responses to pathogens is the expression of C-type lectin receptors (CLRs). Different signaling pathways are triggered by CLRs following the binding of pathogens, which are responsible for triggering the expression of specific cytokines that determine the fate of T cells during polarization. The activation of certain CLRs can be accompanied by the activation of nuclear factor-kappa B, while other CLRs can influence the activation of Toll-like receptors via signaling pathways. Depending on what signaling motifs are present in the cytoplasmic domains of CLRs, they can induce many different types of responses, including pro-inflammatory, antimicrobial, endocytic, phagocytic, and anti-inflammatory responses [68]. Th17 cells have been found to belong to a subgroup of cells that secrete IL-17, or IL-17A, a component of the inflammatory response. Together with Thl, Th2, and Tregs, Th17 cells make up four subsets of CD4+T cells. Under the stimulation of IL-6 and TGF-β, Th17 cells are differentiated by Th0 cells. A key role that they play is in the regulation of the immune system and in the defense of the host [69]. One of the most important transcription factors in maintaining oxygen homeostasis is hypoxia-inducible factor 1 (HIF-1), which is one of the many transcription factors involved in the process. There are two subunits of this protein: an inducibly expressed HIF-1alpha subunit and a constitutively expressed HIF-1beta subunit. In the presence of normoxia, HIF-1 alpha undergoes a process of hydroxylation at specific prolyl residues in order to undergo an immediate ubiquitination and subsequently be degraded by the proteasome. Contrary to this, under hypoxia, the alpha subunit of HIF-1 becomes stable and begins to interact with coactivators such as p300/CBP in order to modulate its transcriptional activity. As a master regulator of hypoxia-inducible genes, HIF-1 regulates a number of hypoxia-inducible genes under hypoxic conditions. The HIF-1 gene family encodes proteins that play a key role in improving oxygen delivery and enhancing cells’ adaptive responses to oxygen deprivation. It is important to note that nitric oxide and several growth factors are also stimulatory factors that can induce HIF-1, so it is not only in response to decreased oxygen availability that it is induced but also in response to other stimulants [70].

Furthermore, we performed docking experiments for the biosurfactants and the ten Hub genes in accordance with the “compounds-targets networks”. Additionally, the results of docking analyses confirmed our results and showed that lichenysin, iturin, surfactin, rhamnolipid, subtilisin, and polymyxin bind stably to the active pockets of target proteins. Therefore, these compounds could be considered for use as a potential treatment for listeriosis by inhibiting proteins such as, IL2, MAPK1, SRC, EGFR, PTPRC, TNF, IL1B, and ERBB2. Taking into account the role of network pharmacology, the present study examines the active biosurfactants, their potential targets, and their associated pathways, as they pertain to the treatment of listeriosis, which provides a theoretical foundation for further experimental studies. In consideration of the limitations of network pharmacology, it is only through data mining that the basic pharmacological mechanisms for the treatment of listeriosis can be identified. Currently, network pharmacology relies on a variety of databases to support the analysis of bioactive properties. Due to the fact that there are many different information sources and experimental data in databases, it is inevitable that they will show discrepancies. In spite of the fact that we have presented some interesting results, further research and clinical trials are required to evaluate the potential of biosurfactants to validate their usage as a prevention measure against *Listeria* and other food-borne diseases.

## 4. Materials and Methods

### 4.1. Biosurfactants Target Prediction

In the present study, we selected biosurfactants that have been reported to possess antimicrobial activity in the literature. The information about their structure, molecular weights, and canonical smiles and the corresponding sdf files were obtained from the PubChem database (http://pubchem.ncbi.nlm.nih.gov/), accessed on 13 September 2022. Using a public database, SwissTargetPrediction, and a STITCH database, we predicted the target of bioactive microbial biosurfactants based on the species of *Homo sapiens* as the only species targeted for the study. To complete the process of standardizing the target names, UniProtKB database (https://www.uniprot.gov/) was used [71,72].

### 4.2. Network Construction for Compound–Targets

The biosurfactants that were collected and the effective targets that were identified were analyzed by using Cytoscape 3.9.1 software (http://www.cytoscape.org/) for the creation of a compound–target network. To measure the topology scores of the nodes in the network, we used the CytoNCA plugin (v2.1.6), which measures betweenness, closeness, and the centrality of subgraphs of the nodes in each graph. Accordingly, the option “without weight” was selected [73]. 

### 4.3. Protein Targets Associated with Listeriosis

A search for targets related to listeriosis was conducted by using keywords such as “listeriosis” and “listeriosis infection” in the GeneCards database (https://www.genecards.org/) [74], Online Mendelian Inheritance in Man database (OMIM, https://omim.org/), and gene–disease associations database (DisGeNET, http://www.disgenet.org/) accessed on 20 September 2022 [75,76], and the Universal Protein database (UniProt, https://www.UniProt.org/) was used to convert the target protein name to a gene name [77]. All listeriosis targets were acquired after repetitive targets were removed.

### 4.4. Target Screening and Network Construction for Biosurfactants and Listeriosis

In order to detect the core target of the biosurfactants for the treatment of listeriosis, the prediction results of the biosurfactants were matched with the search results of the listeriosis related targets, and the target with the most overlap was selected as the core target. Using the FunRich Tool version 3.1.3, we mapped the targets that biosurfactants and listeriosis share. A Venn diagram was drawn in order to visualize the process. In order to construct a common target network, the Cytoscape software version 3.9.1 was used.

### 4.5. Protein–Protein Interaction Network (PPI) Construction and Target Identification

The GeneMANIA tool, in addition to constructing a PPI network, is able to find a series of genes related to the input gene based on a large volume of function-related data and analyze the interaction between these genes, based on their co-localization and co-expression [78]. In the present study, GeneMANIA was used to build a protein–protein interaction network related to the cross-gene interactions between biosurfactants and listeriosis based on the analysis of the cross-gene analysis. As a result of the GeneMANIA analysis, we were able to obtain not only information about the relationships between the input cross genes, but also information about the relationships between other closely related targets as well. Accordingly, we label this new set of genes predicted to be biosurfactant targets for listeriosis in the following analysis. The topology parameters of the PPI network were calculated by using Network Analyzer in order to identify the main nodes of the network and the key proteins across the network, while the degree of centrality of the network (betweenness, closeness, and subgraph) was calculated by using CytoNCA.

### 4.6. Analysis of Gene Ontology (GO) Function and Kyoto Encyclopedia of Genes and Genomes (KEGG) Pathway Enrichment

Based on the results obtained from the above screening, the target of biosurfactants that shared a common target with listeriosis was imported into the Enrichr database (https://maayanlab.cloud/Enrichr/). An analysis was conducted to explore the enrichment of GO functions and pathways within the human genome based on the species *H. sapiens*. As part of the functional analysis of GO, we considered biological processes (BPs), cellular components (CCs), and molecular functions (MFs). The data were visualized as histograms and bubble charts, using the SRPLOT application (http://bioinformatics.com.cn/srplot), as well as the ShinyGO 0.76.2 database (https://bioinformatics.sdstate.edu/go/).

### 4.7. Construction of Target-Path/Functional Networks

In order to perform a deeper analysis of the signal pathways, biological processes, and molecular functions, ten representative pathways were screened. As part of the construction of the target pathway/functional network, the ShinyGO 0.76.2 database was used (https://bioinformatics.sdstate.edu/go/). Through the use of enrichment analysis, potential targets of biosurfactants for treating listeriosis, biological processes, and signaling pathways were defined by nodes in the network, and the interactions between these nodes were defined by edges.

### 4.8. Findings of Hub Genes 

We tested the PPI network obtained from STRING, using the CytoHubba plugin of Cytoscape. This plugin was used to analyze the core regulatory genes of the PPI network, as well as the identification of key targets within the network. As part of the screening process, the core compounds were tested under the assumption that the “Degree” parameter of the node in the “active ingredient target-disease” network was above the mean. A virtual screening approach based on molecular docking was carried out between the biosurfactants and identified hub genes. 

### 4.9. Molecular Docking Analysis

#### 4.9.1. Protein and Ligand Structures

To study the interaction of selected biosurfactants with the identified protein targets in the present study, a molecular docking analysis was performed. Crystal structures of identified hub target proteins such as TNF (PDB ID: 2AZ5), EGFR (PDB ID: 4WKQ), IL1B (PDB ID: 9ILB), IL2 (PDB ID: 1M4C), SRC (PDB ID: 4MXO), PTPRC (PDB ID: 5FMV), ITGB1 (PDB ID: 7CEB), ERBB2 (PDB ID: 3WLW), MTOR (PDB ID: 5WBU), and MAPK1 (PDB ID: 4G6O) were retrieved from RCSB PDB. Two-dimensional structures of selected biosurfactants were retrieved from the well-known organic compound database PubChem in SDF format. These compounds were then converted into three-dimensional structures, using Avogadro, and saved in PDB format [79].

#### 4.9.2. Ligand Preparation

For the preparation of input files for docking, Autodock software 1.5.7 [80] was used. The structures were minimized with MMFF94 force field. Steepest Descent algorithm was used for optimization with a total of 5000 steps. During minimization, the structure was updated every 1 step, and minimization was terminated when the energy difference is less than 0.1. Energy-minimized structures were saved in PDB format.

#### 4.9.3. Prediction of Binding Site 

The binding sites of all protein structures were predicted by using Discovery Studio v. 21.1.0.20298 [81]. The pocket with the highest score was considered to be the most probable binding site of the proteins.

#### 4.9.4. Molecular Docking

Three-dimensional structures of proteins derived from RCSB PDB were prepared for molecular docking, using AutoDock Tools [80]. Before the docking experiment, we used AutoDock Tools software to preprocess the crystal structure of the target proteins, including removing excess protein chains, ligands, and water molecules, and structures were optimized by adding missing hydrogen atoms. Structure files (PDB format) of all biosurfactants were docked separately against the protein structures, using molecular docking software AutoDock 4.2.6. [80]. All the parameters used for the docking of biosurfactants with the proteins were kept the same, except for the grid center differed for each protein inside the grid box. Auto Grid was used for the preparation of the grid map, using a grid box. The grid size was set to 90 × 90 × 90xyz points for all proteins. Grid spacing was kept to 0.500 Å for all the proteins. The grid center for 2AZ5 was designated at dimensions (x, y, and z), −14.888, 68.771, and 32.730; for 4WKQ at (x, y, and z), −2.761, 201.806, and 26.195; for 9ILB at (x, y and z), −13.592, 13.466, and 0.200; for 1M4C at (x, y, and z), 14.170, −10.108, and 20.056; for 4MXO at (x, y, and z), 9.193, −33.735, and −7.984; for 5MFV at (x, y, and z), 30.052, −18.946, and 32.074; for 7CEB at (x, y, and z), 43.112, 46.089, and −1.124; for 3WLW at (x, y, and z), 36.088, 26.277, and −20.954; for 5WBU at (x, y, and z), 11.014, −18.240, and −30.383; and for 4G6O at (x, y, and z), 14.790, 5.794, and 17.485. The grid box is cantered in such a way that it encloses the entire binding site of each protein and provides enough space for the translation and rotation of ligands. The generated docked conformation was ranked by predicted binding energy, and the topmost binding energy docked conformation was analyzed through the use of the PyMOL and Discovery Studio Visualizer [81]. By using the Discovery Studio Visualizer, it was possible to explore the types of interactions, the participating residuals, and the atomic coordinates involved.

## 5. Conclusions

The purpose of this study was to investigate the molecular mechanisms of biosurfactants to treat listeriosis, using a network pharmacology approach and molecular docking. As a result of the current study, biosurfactants have been found to be capable of targeting multiple proteins and regulating multiple signaling pathways induced by *L. monocytogenes* infection, indicating that biosurfactants may have a regulatory effect on listeriosis caused by *L. monocytogenes*. Furthermore, our findings indicate that IL2, MAPK1, EGFR, PTPRC, TNF, ITGB1, IL1B, ERBB2, and mTOR genes may be viable therapeutic targets for the reduction of listeriosis. In addition to providing an alternative or complementary therapy for the treatment of listeriosis, these findings lay the foundation for future studies. There are, however, some limitations to this study, as pharmacological and clinical research still needs to be conducted to verify our findings. A groundwork has been laid for further study of biosurfactants’ protective mechanisms and drug discovery applications based on network pharmacology.

## Figures and Tables

**Table 1:** List of biosurfactants.

Sr. No.	Biosurfactant	Microbial Origin	References	Molecular Formula	PubChem	Canonical SMILE
1	Surfactin	*Bacillus subtilis* *Bacillus siamensis*	[19,20]	C_53_H_93_N_7_O_13_	5066078	CC(C)CCCCCCCCCC1CC(=O)NC(C(=O)NC(C(=O)NC(C(=O)NC(C(=O)NC(C(=O)NC(C(=O)NC(C(=O)O1)CC(C)C)CC(C)C)CC(=O)O)C(C)C)CC(C)C)CC(C)C)CCC(=O)O
2	Rhamnolipid	*Pseudomonas aeruginosa*	[21]	C_32_H_58_O_13_	5458394	CCCCCCCC(CC(=O)O)OC(=O)CC(CCCCCCC)OC1C(C(C(C(O1)C)O)O)OC2C(C(C(C(O2)C)O)O)O
3	Viscosin	*Pseudomonas fluorescens*	[22]	C_54_H_95_N_9_O_16_	72937	CCCCCCCC(CC(=O)NC(CC(C)C)C(=O)NC(CCC(=O)O)C(=O)NC1C(OC(=O)C(NC(=O)C(NC(=O)C(NC(=O)C(NC(=O)C(NC(=O)C(NC1=O)C(C)C)CC(C)C)CO)CC(C)C)CO)C(C)CC)C)O
4	Liposan	*Candida lipolytica*	[23]	C_8_H_14_O_2_S_2_	864	C1CSSC1CCCCC(=O)O
5	Lichenysin	*Bacillus licheniformis*	[24]	C_51_H_90_N_8_O_12_	11804102	CC(C)CCCCCCCCC1CC(=O)NC(C(=O)NC(C(=O)NC(C(=O)NC(C(=O)NC(C(=O)NC(C(=O)NC(C(=O)O1)C(C)C)CC(C)C)CC(=O)O)C(C)C)CC(C)C)CC(C)C)CCC(=O)N
6	Iturin	*Bacillus subtilis* *Bacillus amyloliquefaciens*	[25]	C_48_H_74_N_12_O_14_	158570	CCCCCCCCCCCC1CC(=O)NC(C(=O)NC(C(=O)NC(C(=O)NC(C(=O)N2CCCC2C(=O)NC(C(=O)NC(C(=O)N1)CO)CC(=O)N)CCC(=O)N)CC(=O)N)CC3=CC=C(C=C3)O)CC(=O)N
7	Arthrofactin	*Arthrobacter* sp. strain MIS38	[26]	C_64_H_111_N_11_O_20_	23724538	CCCCCCCC1CC(=O)NC(C(=O)NC(C(=O)NC(C(=O)NC(C(=O)NC(C(=O)NC(C(=O)NC(C(=O)NC(C(=O)NC(C(=O)NC(C(=O)NC(C(=O)O1)CC(=O)O)C(C)CC)C(C)CC)CO)CC(C)C)CO)CC(C)C)CC(C)C)C(C)O)CC(=O)O)CC(C)C
8	Amphisin	*Pseudomonas* *fluorescens*	[27]	C66H114N12O20	101134740	CCCCCCCC(CC(=O)NC(CC(C)C)C(=O)NC(CC(=O)O)C(=O)NC1C(OC(=O)C(NC(=O)C(NC(=O)C(NC(=O)C(NC(=O)C(NC(=O)C(NC(=O)C(NC(=O)C(NC1=O)CC(C)C)CC(C)C)CO)CC(C)C)CCC(=O)N)CC(C)C)C(C)CC)CC(=O)O)C)O
9	Putisolvin	*Pseudomonas putida*	[28]	C65H113N13O19	139588800	CCCCCC(=O)NC(CC(C)C)C(=O)NC(CCC(=O)O)C(=O)NC(CC(C)C)C(=O)NC(C(C)CC)C(=O)NC(CCC(=O)N)C(=O)NC(CO)C(=O)NC(C(C)C)C(=O)NC(C(C)CC)C(=O)NC1COC(=O)C(NC(=O)C(NC(=O)C(NC1=O)CC(C)C)C(C)C)CO
10	Ustilagic Acid	*Ustilago maydis*	[29]	C36H64O18	52922086	CCCC(CC(=O)OC1C(C(C(OC1OC2C(OC(C(C2O)O)OCC(CCCCCCCCCCCCC(C(=O)O)O)O)COC(=O)C)CO)O)O)O
11	Pumilacidin	*Bacillus pumilus*	[30]	C55H99N7O12	101174694	CCC(C)C1C(=O)OC(CC(=O)NC(C(=O)NC(C(=O)NC(CNC(C(=O)NC(C(=O)NC(C(=O)N1)CC(C)C)CC(=O)O)CC(C)C)CC(C)C)CC(C)C)CCC(=O)O)CCCCCCCCCCC(C)C
12	Fengycin	*Bacillus subtilis*	[25]	C72H110N12O20	443591	CCCCCCCCCCCCCC(CC(=O)NC(CCC(=O)O)C(=O)NC(CCCN)C(=O)NC1CC2=CC=C(C=C2)OC(=O)C(NC(=O)C(NC(=O)C(NC(=O)C3CCCN3C(=O)C(NC(=O)C(NC(=O)C(NC1=O)C(C)O)CCC(=O)O)C)CCC(=O)N)CC4=CC=C(C=C4)O)C(C)CC)O
13	Subtilisin	*Bacillus subtilis*	[31]	C18H25N3O6	92174084	CC(C(=O)NOC(=O)C1=CC=CC=C1)NC(=O)C(C)NC(=O)OC(C)(C)C
14	Gramicidin S	*Brevibacillus brevis*	[32]	C60H92N12O10	73357	CC(C)CC1C(=O)NC(C(=O)N2CCCC2C(=O)NC(C(=O)NC(C(=O)NC(C(=O)NC(C(=O)N3CCCC3C(=O)NC(C(=O)NC(C(=O)N1)CCCN)C(C)C)CC4=CC=CC=C4)CC(C)C)CCCN)C(C)C)CC5=CC=CC=C5
15	Polymyxin	*Paenibacillus polymyxa*	[33]	C48H82N16O14	3083714	CC(C)CC1C(=O)NC(C(=O)NC(C(=O)NC(C(=O)NCCC(C(=O)NC(C(=O)NC(C(=O)N1)CC2=CC=CC=C2)CCN)NC(=O)C(CCN)NC(=O)C(C(C)O)NC(=O)C(CCN)NC(=O)O)C(C)O)CCN)CCN

**Table 2:** Topological parameters of the compound.

Sr. No.	Biosurfactant	Degree	Betweenness	Closeness
1	Putisolvin	12	218.86357	0.44347826
2	Surfactin	11	260.51117	0.42857143
3	Lichenysin	11	180.90701	0.43589744
4	Arthrofactin	10	295.14645	0.4214876
5	Amphisin	10	98.94803	0.4214876
6	Iturin	10	312.37665	0.39534885
7	Pumalicidin	10	114.981255	0.42857143
8	Subtilisin	10	543.5165	0.39534885
9	Polymyxin	9	186.33307	0.4015748
10	Viscosin	9	126.53945	0.4214876
11	Fengycin	8	143.53413	0.38345864
12	Gramicidin	8	158.42682	0.38345864
13	Ustilagic Acid	6	220.03003	0.3167702
14	Rhamnolipid	6	151.10707	0.37226278
15	Liposan	3	112.77881	0.3090909

**Table 3:** Topological parameters of the targeted proteins.

Sr. No.	Genes	Degree	Betweenness	Closeness
1	TNF	27	157.15123	0.24
2	EGFR	26	212.85284	0.23841059
3	SRC	21	59.026463	0.23076923
4	IL2	21	49.672054	0.23076923
5	IL1B	21	72.07377	0.23076923
6	PTPRC	19	35.360935	0.2264151
7	ITGB1	17	31.785282	0.22360249
8	ERBB2	17	19.92101	0.225
9	MAPK1	15	20.884993	0.22222222
10	MTOR	15	14.559942	0.22222222
11	MDM2	14	31.841478	0.2208589
12	ITGB2	13	11.806349	0.21818182
13	HDAC1	13	26.021725	0.2195122
14	NR3C1	12	25.276262	0.21818182
15	TERT	12	2.5834055	0.21818182
16	MET	11	4.9985447	0.21686748
17	SELL	11	11.046661	0.21301775
18	CASP1	9	2.6095238	0.21301775
19	HDAC6	9	5.4996777	0.21301775
20	NOD2	8	3.3137822	0.20930232
21	SELE	8	0	0.21176471
22	SELP	8	0	0.21176471
23	ADAM17	7	1.1746032	0.21052632
24	SIRT2	7	2.0468254	0.20571429
25	ITK	7	6.074603	0.20809248
26	HDAC2	7	4.533211	0.20571429
27	PTPN2	6	2.3246753	0.20571429
28	ITGB7	5	0.22222222	0.2
29	PLA2G2A	5	0.2	0.20809248
30	UBE2I	4	0	0.18947369
31	PPIA	4	1.137931	0.20224719
32	MAP3K8	3	0	0.20454545
33	IGF2R	1	0	0.19672132
34	OPRD1	1	0	0.027777778
35	SLC5A1	1	0	0.19672132
36	F11	1	0	0.027777778
37	MRGPRX1	0	0	0.027027028

**Table 4:** Interactive active site residues top-rated pose of biosurfactants with target proteins.

Sr. No.	Protein	Receptor–Ligand	Interaction Type	Distance
1	1M4C	A:ARG83:HN2 - :UNL1:O	Conventional Hydrogen Bond	2.07493
		UNL1:H - :UNL1:O	Conventional Hydrogen Bond	1.65486
		UNL1:H - :UNL1:O	Conventional Hydrogen Bond	1.62878
		A:MET23 - :UNL1	Alkyl	5.36375
		UNL1 - A:MET23	Alkyl	5.22848
		UNL1 - A:LEU85	Alkyl	4.12048
2	3WLW	A:TYR268:HH - N:UNK1:O	Conventional Hydrogen Bond	2.03597
		N:UNK1:H - A:ASP286:OD1	Conventional Hydrogen Bond	2.62308
		N:UNK1:H - N:UNK1:O	Conventional Hydrogen Bond	2.48544
		N:UNK1:H - N:UNK1:O	Conventional Hydrogen Bond	2.1777
		N:UNK1:H - N:UNK1:O	Conventional Hydrogen Bond	2.83049
		N:UNK1:H - N:UNK1:O	Conventional Hydrogen Bond	1.55546
		N:UNK1:H - N:UNK1:O	Conventional Hydrogen Bond	2.51507
		N:UNK1:H - A:THR285:O	Conventional Hydrogen Bond	2.22765
		N:UNK1:H - A:SER284:O	Conventional Hydrogen Bond	3.005
		N:UNK1:H - N:UNK1:O	Conventional Hydrogen Bond	2.72822
		A:LYS309:CE - N:UNK1:O	Carbon Hydrogen Bond	3.69856
		A:LEU250:CB - N:UNK1	Pi-Sigma	3.92346
		A:ALA249 - N:UNK1	Alkyl	4.39901
		A:ALA249 - N:UNK1:C	Alkyl	4.10378
		A:VAL251 - N:UNK1	Alkyl	5.23527
		A:VAL251 - N:UNK1	Alkyl	5.01063
		N:UNK1 - A:LEU250	Alkyl	4.96966
		N:UNK1:C - A:VAL287	Alkyl	5.09767
3	4MXO	A:MET341:HN - N:UNK1:O	Conventional Hydrogen Bond	1.97267
		A:SER345:HN - N:UNK1:O	Conventional Hydrogen Bond	2.85586
		A:ASN391:HD21 - N:UNK1:O	Conventional Hydrogen Bond	2.986
		A:ASN391:HD22 - N:UNK1:O	Conventional Hydrogen Bond	2.8606
		N:UNK1:H - A:LEU273:O	Conventional Hydrogen Bond	2.23507
		N:UNK1:H - A:LEU273:O	Conventional Hydrogen Bond	2.69853
		N:UNK1:H - A:GLN275:O	Conventional Hydrogen Bond	2.83421
		A:GLY274:CA - N:UNK1:O	Carbon Hydrogen Bond	3.14193
		A:GLY344:CA - N:UNK1:O	Carbon Hydrogen Bond	3.14994
		N:UNK1:C - N:UNK1:O	Carbon Hydrogen Bond	3.54737
		A:VAL281 - N:UNK1	Alkyl	4.86428
		A:VAL281 - N:UNK1	Alkyl	5.14814
		A:ALA293 - N:UNK1	Alkyl	4.50016
		A:LYS295 - N:UNK1	Alkyl	5.25134
		A:ALA403 - N:UNK1:C	Alkyl	3.80138
		N:UNK1:C - A:MET314	Alkyl	4.94124
		N:UNK1:C - A:VAL323	Alkyl	3.63942
		N:UNK1 - A:LEU273	Alkyl	4.79106
		N:UNK1:C - A:LEU273	Alkyl	4.84683
		A:PHE278 - N:UNK1	Pi-Alkyl	5.36838
4	5FMV	N:UNK1:H - A:ASP508:OD2	Salt Bridge	2.60084
		N:UNK1:H - A:ASP508:OD2	Conventional Hydrogen Bond	2.42554
		N:UNK1:H - A:ASP508:OD1	Conventional Hydrogen Bond	2.71193
		A:LYS448 - N:UNK1	Alkyl	4.81167
		A:PRO449 - N:UNK1	Alkyl	4.98618
		A:HIS404 - N:UNK1	Pi-Alkyl	4.73078
		A:TRP487 - N:UNK1	Pi-Alkyl	4.80377
		A:TRP487 - N:UNK1	Pi-Alkyl	4.83494
		A:TRP487 - N:UNK1	Pi-Alkyl	4.37643
5	9ILB	N:UNK1:H - A:THR79:OG1	Conventional Hydrogen Bond	2.04809
		N:UNK1:H - N:UNK1:O	Conventional Hydrogen Bond	2.48545
		N:UNK1:H - A:GLU25:OE2	Conventional Hydrogen Bond	3.01317
		N:UNK1:H - N:UNK1:O	Conventional Hydrogen Bond	2.08891
		N:UNK1:H - N:UNK1:O	Conventional Hydrogen Bond	1.5553
		N:UNK1:H - A:LEU134:O	Conventional Hydrogen Bond	2.12807
		N:UNK1:H - A:VAL132:O	Conventional Hydrogen Bond	2.61684
		N:UNK1:H - A:LEU80:O	Conventional Hydrogen Bond	2.59836
		N:UNK1:C - N:UNK1:O	Carbon Hydrogen Bond	3.5902
		A:PHE133 - N:UNK1	Pi-Pi Stacked	3.75995
		A:TYR24 - N:UNK1	Pi-Alkyl	4.38175
		A:TYR24 - N:UNK1:C	Pi-Alkyl	4.05141
		N:UNK1 - A:PRO131	Pi-Alkyl	5.36192
6	4G6O	A:ASN152:HD22 - :UNL1:N	Conventional Hydrogen Bond	2.68599
		UNL1:H - :UNL1:O	Conventional Hydrogen Bond	1.97392
		UNL1:H - :UNL1:O	Conventional Hydrogen Bond	1.72687
		UNL1:H - :UNL1:O	Conventional Hydrogen Bond	2.6197
		UNL1:H - A:ASP109:OD2	Conventional Hydrogen Bond	2.68182
		UNL1:H - :UNL1:O	Conventional Hydrogen Bond	2.16901
		UNL1:H - A:CYS164:SG	Conventional Hydrogen Bond	3.02337
		UNL1:H - :UNL1:O	Conventional Hydrogen Bond	2.8299
		UNL1:H - A:ASP104:O	Conventional Hydrogen Bond	2.77312
		UNL1:H - :UNL1:O	Conventional Hydrogen Bond	2.25806
		UNL1:H - :UNL1:O	Conventional Hydrogen Bond	2.61859
		UNL1:H - A:GLU31:OE1	Conventional Hydrogen Bond	2.17908
		UNL1:H - A:ASN152:OD1	Conventional Hydrogen Bond	2.75093
		UNL1:H - :UNL1:O	Conventional Hydrogen Bond	2.45343
		UNL1:H - A:ASP165:OD1	Conventional Hydrogen Bond	2.30444
		UNL1:H - :UNL1:O	Conventional Hydrogen Bond	2.29142
		UNL1 - A:ARG65	Pi-Alkyl	5.08193
7	4WKQ	UNL1:H - A:ASN842:OD1	Conventional Hydrogen Bond	2.34513
		UNL1:H - A:ASP837:OD2	Conventional Hydrogen Bond	2.64181
		UNL1:H - :UNL1:O	Conventional Hydrogen Bond	2.7793
		UNL1:H - :UNL1:O	Conventional Hydrogen Bond	2.61272
		A:ARG841:CD - :UNL1:O	Carbon Hydrogen Bond	3.48781
		UNL1:C - A:ASP855:OD2	Carbon Hydrogen Bond	3.36649
		UNL1:C - A:ASP855:OD2	Carbon Hydrogen Bond	2.93591
		A:LEU718 - :UNL1	Alkyl	4.75503
		A:LEU718 - :UNL1	Alkyl	4.63701
		A:VAL726 - :UNL1	Alkyl	4.56822
		A:VAL726 - :UNL1	Alkyl	5.49427
		A:ALA743 - :UNL1	Alkyl	4.47905
		A:LEU844 - :UNL1	Alkyl	5.36797
		A:LEU844 - :UNL1	Alkyl	5.0695
		UNL1:C - A:LYS745	Alkyl	4.04982
		UNL1:C - A:MET766	Alkyl	5.00922
		UNL1:C - A:LEU788	Alkyl	4.51178
8	5WBU	UNL1:H - A:MET2345:SD	Conventional Hydrogen Bond	2.81877
		A:ILE2356:CG2 - :UNL1	Pi-Sigma	3.70617
		A:TYR2225 - :UNL1	Pi-Pi T-shaped	4.91263
		UNL1:C - A:PRO2169	Alkyl	4.70722
9	7CEB	A:TYR295:HH - :UNL1:O	Conventional Hydrogen Bond	2.30825
		A:TYR411:HH - :UNL1:O	Conventional Hydrogen Bond	2.74757
		UNL1:H - :UNL1:O	Conventional Hydrogen Bond	1.65568
		UNL1:H - :UNL1:O	Conventional Hydrogen Bond	1.6278
		UNL1:C - A:TYR234	Pi-Sigma	3.72179
		UNL1:C - A:ILE356	Alkyl	4.29736
		UNL1:C - A:PRO185	Alkyl	4.4092
		A:TRP91 - :UNL1	Pi-Alkyl	5.11969
		A:TRP91 - :UNL1	Pi-Alkyl	5.3081
		A:HIS110 - :UNL1	Pi-Alkyl	5.20706
		A:PHE237 - :UNL1	Pi-Alkyl	5.3189
		A:PHE237 - :UNL1	Pi-Alkyl	4.60088
		A:TYR295 - :UNL1	Pi-Alkyl	4.91972
		A:TYR295 - :UNL1:C	Pi-Alkyl	5.27292
		A:TYR411 - :UNL1:C	Pi-Alkyl	4.32441
10	2AZ5	A:GLN61:HE12 - :UNL1:O	Conventional Hydrogen Bond	2.61257
		A:TYR119:HH - :UNL1:O	Conventional Hydrogen Bond	2.86757
		A:TYR151:HH - :UNL1:O	Conventional Hydrogen Bond	2.59785
		A:LEU63:CD1 - :UNL1	Pi-Sigma	3.80396
		A:LEU63:CD2 - :UNL1	Pi-Sigma	3.73556
		UNL1:C - A:TYR119	Pi-Sigma	3.95427
		UNL1 - A:PRO117	Pi-Alkyl	4.8335

## Data Availability

All data generated or analyzed during this study are included in this article.
